# DNA methylation analysis reveals an epigenetic signature distinctive of high-grade oligodendroglioma

**DOI:** 10.1007/s00401-025-02926-y

**Published:** 2025-08-23

**Authors:** Katharina Johanna Weber, Mareike Dettki, Marco Münzberg, Pia Susann Zeiner, Marie-Thérèse Forster, Eike Steidl, Iris Divé, Patrick Nikolaus Harter, Michael Scherer

**Affiliations:** 1 Goethe University Frankfurt, University Hospital, Neurological Institute (Edinger Institute), Heinrich-Hoffmann-Straße 7, 60528 Frankfurt Am Main, Germany; 2 German Cancer Consortium (DKTK), Germany and German Cancer Research Center (DKFZ), Im Neuenheimer Feld 280, Heidelberg, 69120 Germany; 3 Frankfurt Cancer Institute (FCI), Paul-Ehrlich-Str. 42-44, 60596 Frankfurt Am Main, Germany; 4 Goethe University Frankfurt, University Hospital, University Cancer Center (UCT) Frankfurt, Theodor-Stern-Kai 7, 60590 Frankfurt am Main, Germany; 5 Goethe University Frankfurt, University Hospital, Dr. Senckenberg Institute of Neurooncology, Schleusenweg 2-16, 60528 Frankfurt Am Main, Germany; 6 Goethe University Frankfurt, University Hospital, Department of Neurology, Schleusenweg 2-16, 60528 Frankfurt Am Main, Germany; 7 Goethe University Frankfurt, University Hospital, Department of Neurosurgery, Schleusenweg 2-16, 60528 Frankfurt Am Main, Germany; 8 Goethe University Frankfurt, University Hospital, Institute of Neuroradiology, Schleusenweg 2-16, 60528 Frankfurt Am Main, Germany; 9 German Cancer Consortium (DKTK), Partner Site Munich, A Partnership Between German Cancer Research Center (DKFZ), Im Neuenheimer Feld 280, 69120 Heidelberg, Germany; 10 Center for Neuropathology and Prion Research, Faculty of Medicine, Ludwig Maximilians University Munich, University Hospital, Feodor-Lynen-Straße 23, 81377 Munich, Germany; 11 Bayerisches Zentrum Für Krebsforschung (BZKF), Partner Site Munich, Marchioninistraße 15, 81377 Munich, Germany; 12 University Hospital, Ludwig Maximilians University Munich, Geschwister-Scholl-Platz 1, 80539 Munich, Germany; 13 Divison of Cancer Epigenomics, German Cancer Research Center (DKFZ), Im Neuenheimer Feld 280, 69120 Heidelberg, Germany

**Keywords:** IDH mutant glioma, Epigenetic brain tumor classification, Latent methylation component, CNS WHO grading, Oligodendroglioma

## Abstract

IDH-mutant gliomas represent a subtype of diffuse gliomas that primarily affect patients in early to mid-adolescence. These tumors are classified into three distinct CNS WHO grades of malignancy. Accurate grading is essential for selecting an appropriate treatment maximizing anti-tumor efficacy while minimizing adverse effects. However, grading of oligodendrogliomas with 1p/19q codeletion currently relies on qualitative tumor characteristics that may be influenced by observer subjectivity, sampling bias, and tumor heterogeneity. This study aimed to explore DNA methylation-based tumor deconvolution into latent methylation components (LMCs) to evaluate their potential as objective grading tools in a cohort of 137 IDH-mutant gliomas. LMCs were analyzed in relation to malignancy markers, cellular composition, and underlying methylation signatures of the chromatin landscape. Glioma subtypes were associated with distinct LMCs. Two LMCs correlated with higher cellular density and advanced epigenetic age as well as with microvascular proliferation, necrosis, and epigenetically defined high-grade astrocytoma. The epigenetic patterns defining high-grade astrocytoma or oligodendroglioma, respectively, were similar. Higher-grade oligodendrogliomas, identified by LMC-based grading, were associated with more copy number alterations. Among patients of an external cohort who died during the assessment period, higher LMC1 proportions were associated with poorer overall survival. Therefore, LMCs hold the potential to support IDH-mutant glioma grading by incorporating objective epigenetic markers.

**Supplementary Information:**

The online version contains supplementary material available at 10.1007/s00401-025-02926-y.

## Introduction

Isocitrate dehydrogenase mutant (IDHmt) gliomas belong to adult-type diffuse gliomas and display an average annual age-adjusted incident rate of 0.74/100.000 [32]. Codeleted oligodendroglioma can be distinguished from non-codeleted astrocytoma based on aberrations of chromosomal arms 1p and 19q [23]. According to the classification of central nervous system (CNS) tumors by the WHO, the highest CNS WHO grade, 4, is assigned to astrocytomas with a homozygous deletion of the cell cycle checkpoint *CDKN2A/B*, tumor necrosis, or microvascular proliferation [23]. Neuropathological grading of lower-grade astrocytomas and oligodendrogliomas in general is more challenging, especially since definitive molecular features are lacking. Whereas astrocytomas without clear features of malignancy but increased mitotic activity, marked nuclear pleomorphism, or high cellular density are graded as CNS WHO 3, grade 2 astrocytoma lacks pleomorphism, brisk mitotic figures, and increased cellularity [23]. With these qualitative histological traits, the boundaries between the assignment of lower grades can be blurred and observer dependent. For oligodendroglioma, of which CNS WHO grade 2 and 3 are recognized, grading is even more demanding [16]. While higher cellularity and marked nuclear atypia are majorly found in higher-grade oligodendroglioma, the presence of microvascular proliferation and/or necrosis is not strictly considered as criteria for CNS WHO grade 3 [23]. Prior studies provided evidence that homozygous deletion of *CDKN2A/B*, the presence of chromosomal aberrations other than 1p/19q-codeletion, and a brisk mitotic frequency higher than 2.5 mitoses/mm^2^ might be associated with higher-grade tumors [1, 9, 11, 16]. From an epidemiological perspective, two age peaks of oligodendroglioma incidence were observed and increased age at first diagnosis was reported as a reliable prognostic factor for lower survival probability [9, 11]. Cellular aging can be inferred from changes in DNA methylation patterns rendering estimates of an “epigenetic age” in comparison to chronological age [18]. Since accelerated epigenetic aging of tumors was associated with 1p/19q-codeletion and a survival benefit for patients suffering from glioblastoma, IDH wildtype, its potential as a prognostic biomarker in gliomas warrants further investigation [3, 5, 22].

IDH mutant gliomas harbor distinct epigenetic alterations, including global DNA hypermethylation through the inhibition of TET demethylases, which is orchestrated via the production of the mutation-induced oncoprotein 2-HG [13, 37, 40]. The increased level of DNA methylation at CpG islands—genomic regions regulating gene expression at promoters—coined the terminology of G-CIMP, Glioma CpG Island Methylator Phenotype [29, 31]. An important milestone was the development of a DNA-methylation based classifier of brain tumors, which improved diagnostic accuracy [6]. Albeit astrocytomas can be differentiated into “astrocytoma” and “high-grade astrocytoma” based on this classifier, this epigenetic graduation is not clearly associated with CNS WHO grades [23]. For oligodendroglioma, furthermore, the brain tumor classifier recognizes one methylation class only without a distinction between higher- and lower-grade methylation signatures. Since DNA methylation-based profiling of primary brain tumors is comprehensively used in diagnostics, the exploration of epigenetic alterations which corroborate CNS WHO grading is very appealing [6, 20]. While recent studies contributed to the refined classification of astrocytomas based on DNA methylation profiles, similar approaches have not been leveraged for oligodendroglioma [15, 35]. Since neuro-oncological patient treatment demands optimized chemotherapeutic strategies, and the clinical use of most recently accessible molecular compounds is restricted to lower-grade gliomas, it is important to further understand malignant transition and to define the CNS WHO grades more precisely [27].

Therefore, we set out for an observer-independent, in-depth investigation of DNA methylation signatures of IDH mutant gliomas using reference-free deconvolution [34]. We show that Latent Methylation Components (LMCs) are associated with glioma subtypes, histologic tumor characteristics, and CNS WHO grades. Focusing on oligodendroglioma, we propose an LMC-based grading, which coalesced with increased chromosomal aberrations and improved the stratification of oligodendroglioma patients in the low-grade glioma cohort provided by the Cancer Genome Atlas (TCGA).

## Materials and methods

### Collection of epidemiological, molecular and neuro-radiological data

Patients treated at the tertiary center for neuro-oncology of the University Hospital Frankfurt for epigenetically classified IDH mutant glioma between 2016 and 2022 were enrolled. Electronic patient records were screened for sex, age at diagnosis, Karnofsky Performance Score (KPS) prior to surgery, extent of resection, and therapy regimen. The MRI scans prior to surgery were reviewed for contrast agent enrichment in the tumors by a trained neuro-radiologist (ES). Patients treated with inhibitors targeting mutant IDH were not included in this study. DNA methylation classes were obtained using the Heidelberg (HD) brain tumor classifier by uploading DNA methylation data onto molecularneuropathology.org. We obtained calibrated scores (v11b4 and v12.5) and MGMT promoter methylation status for each tumor (© MolecularNeuroPathology.org 2023). In addition, visual copy number analyses for assessment of 1p/19q-codeletion status as well as homozygous deletion of the gene locus *CDKN2A/B* were performed based on methylation data. If the estimated copy number profile at the *CDKN2A/B* gene locus was lower than a single chromosomal loss (i.e., a copy number change of 1), a homozygous deletion was assumed [19]. Otherwise, if a drop in the copy number profile was detected for *CDKN2A/B* in 1p/19q-codeleted tumors that did not reach the manual threshold of a homozygous deletion, the deletion was classified as heterozygous [19]. The tumors were classified according to the 5th edition of the WHO classification of Central Nervous System Tumors [23]. The study was approved by the local ethical committee (SNO-4–2023).

### Histology and immunohistochemistry

4 µm-thick tissue sections of Formalin-fixed paraffin-embedded tumor tissue were cut using a microtome (Leica SM 2000R, Germany), mounted on slides (Superfrost Plus, Thermo Scientific, Germany) and stained according to manual H&E routine staining protocols. For samples not originating from stereotaxic biopsy, the H&E slides were evaluated for presence of tumor necroses and microvascular proliferation as well as maximum cellular density using the categories low, intermediate, and high. The mitotic frequency was raised on whole mount H&E stains by careful microscopic analysis of 3 × 10 high power fields and documentation of the highest number of mitotic figures per mm^2^. The immunohistochemical Ki67 staining was performed using the LEICA BOND-III automated stainer (Leica, Wetzlar, Germany) following established protocols (MIB-1, dilution 1:200; DAKO; Glostrup, Denmark). An integrative Ki67-proliferation score was assigned to each whole mount tumor sample. All slides were examined by a board-certified neuropathologist (KJW) using a brightfield microscope (model BX51, field of view 22mm, Olympus; Tokyo, Japan).

### DNA methylation analyses

DNA from punch biopsies of FFPE tissue was extracted using either the Stratek Invisorb Genomic DNA Kit II (stratek molecular, Berlin, Germany) or the Maxwell RSC FFPE Plus DNA Kit (Promega, Madison, Wisconsin, USA) following the manufacturer protocols at the Edinger Institute, University Hospital Frankfurt. DNA concentration was measured using the Qubit DNA BR Assay Kit and Qubit 3 Fluorometer device (Invitrogen, Life Technologies Corporation, Oregon, USA). DNA was processed and hybridized to the Human Methylation EPIC v1 array beadchip (Illumina, San Diego, California, USA) following protocols provided by the manufacturer. The EPIC array beadchips were scanned with an iScan device (Illumina, San Diego, California, USA) and raw intensity data (idats) was generated using the GenomeStudio software (Illumina, San Diego, California, USA). The idats were uploaded onto the website molecularpathology.org provided by the University of Heidelberg, Germany. The idats were furthermore analyzed using the R package “RnBeads” [28], which implements quality control steps including the removal of low-quality CpG probes (including cross-reactive [21]), as well as CpGs in annotated SNPs and on the sex chromosomes. Normalization of methylation data was carried out using the “dasen” method from the R package “watermelon” [33]. The LUMP (“leukocyte unmethylation for purity”) algorithm was used to estimate the overall leukocyte contents from bulk tumor methylomes and uses 44 CpG sites particularly hypomethylated in leukocytes [2, 28].

### DNA methylation-based tumor deconvolution

Reference-free tumor deconvolution of DNA methylation data was performed using “MeDeCom” [34] and the 5000 most variably methylated CpGs. MeDeCom defines major patterns of data variation, called LMCs. One to ten LMCs (parameter: K) were computed and optimal parameter values for K and the regularization parameter lambda (values tested: 0, 0.01, 0.001, 0.0001) were determined using the cross-validation error. Reference-based deconvolution of tumor methylomes was performed with the R package “MethylCIBERSORT” [8]. A reference matrix was built based on two matrices, one from the neoplastic cell fractions from [17] and the second from the non-neoplastic, immune-cell fractions of a reference matrix kindly provided by Tim R Fenton [8]. The reference matrix allows for deriving the cellular composition of a glioma sample through cell type-specific methylation patterns of selected CpG sites according to [8]. Briefly, idats were imported into the R package “minfi” for quality checks, Noob normalization, and acquisition of beta values. The beta value matrices were uploaded onto the CIBERSORT website, and cell type proportions were computed (provided by the Alizadeh Lab, Stanford University, USA, developed by [30].

### Computation of epigenetic tumor ages and DNA methylation age acceleration

For computation of the tumors’ epigenetic ages we used the “RnBeads” R package [28]. DNA methylation age acceleration was calculated by subtracting chronological from epigenetic age.

### Correlation of DNA methylation with CpG island (CGI) relation and chromatin landscape

We determined hyper- vs. hypomethylated sites for each of the LMCs by comparing the DNA methylation states in the LMCs versus the mean of the remaining LMCs using a cutoff of 0.25 as the minimum methylation difference. CpGs are determined as LMC-specifically hypomethylated if their methylation state is less than 0.25 compared to the mean of the other LMCs, and hypermethylated, respectively. For each of the CpGs, we retrieved its University of California Santa Cruz (UCSC) Genome Browser-based gene annotation and CGI relation. The genomic coordinates of each CpG site were mapped to their location within the chromatin landscape by comparison with universal chromatin state annotations of the human genome using a ChromHMM model as described in [38]. Subgroups within each major group of genomic annotations were summarized, like “ReprPC” for original ReprPC subgroups 1–9, for instance.

#### TCGA validation cohort

DNA methylation and meta data from the TCGA low grade glioma (LGG) cohort was downloaded (*n* = 529 cases). Heidelberg Epignostix brain tumor classifier results were collected for the whole TCGA LGG cohort, and re-classification was performed according to the current version of the WHO classification of CNS tumors. Patient reports were reviewed for clinical follow up on patient overall survival for computation of the overall survival probability based on the Kaplan–Meier method. 406 tumors were classifiable as IDH mutant gliomas; 123 tumors were excluded because they were either not classifiable or did not belong to the methylation class of IDH mutant gliomas. 165 tumors showed a 1p/19q-codeletion. Histology reports were reviewed for microvascular proliferation or necrosis, and a CNS WHO grade 3 was assigned if either one was described. CNS WHO grade 3 was assigned if no further information about malignancy signs was available, while the sample was annotated as “anaplastic” in the TCGA documentation. Samples without described necrosis and microvascular proliferation or the annotation “anaplastic” were allocated to CNS WHO grade 2. Additionally, for the 165 gliomas with 1p/19q-codeletion, information about additional copy number alterations (present/absent) and *CDKN2A/B* deletion status (no alteration/heterozygous deletion/homozygous deletion) were collected based on visual inspection of copy number profiles as described for the training cohort. For the 241 IDH mutant gliomas with intact 1p/19q, the methylome-based copy number profiles were reviewed for homozygous deletion of *CDKN2A/B*. If the latter, or necrosis or microvascular proliferation was present, we assigned a CNS grade 4. Astrocytomas without necrosis or microvascular proliferation but the annotation “anaplastic” in the TCGA documentation were assigned to a grade 3; else (i.e. without the annotation “anaplastic”) to a grade 2. TCGA oligodendroglioma patients with a documented event of death were identified by the column “vital status” and were chosen for a sub cohort analysis. The patterns of DNA methylation computed on the Frankfurt training cohort were transferred to the TCGA cases using MeDeCom’s “factorize.regr” function. Based on the information of LMC composition from the training cohort, LMC proportions were analogously computed for the TCGA validation cohort. The 1p/19q-codeleted tumors were dichotomized according to CNS WHO grade and LMC1 proportions less or higher than 0.39.

#### Statistical analysis

Overall survival (OS) was calculated according to the Kaplan–Meier method. Log-Rank p values were computed. All statistical analyses were carried out using JMP18 (SAS, Cary, North Carolina, USA) or R (R Core Team, 2019). The correlation plots were computed with the R package “corrplot” using Pearson correlation coefficients. For figure design, Affinity Designer software was used (version 1.10.6.1665, Serif (Europe) Ltd, Nottingham, UK). The Pearson Chi^2^ test and Likelihood Ratio test were used for categorical variables in contingency tables. For non-parametric data, Wilcoxon, Kruskal–Wallis’ and Dunn’s tests were used. A recursive partitioning model was used with LMC1 proportions as a predictor of the dependent variable (presence or absence of copy number variations (CNVs) in addition to 1p/19q-codeletion in oligodendrogliomas) in the training cohort (*n* = 58, exclusion of *n* = 2 samples due to non-eligible copy number profiles). A p-value below 0.05 was considered statistically significant.

## Results

### Study cohort and workflow

The study cohort comprised 137 patients with IDH mutant gliomas treated in the tertiary center for neuro-oncology at Goethe University Hospital in Frankfurt, Germany. Patient sex (p 0.8601, Fisher’s exact test), Karnofsky Performance Score (KPS; p 0.2650, Wilcoxon), extent of resection (p 0.6901, Pearson) and treatment strategies (p 0.3617, Pearson) were equally distributed among 77 astrocytomas (WHO CNS grade 2–12 (16%), grade 3–39 (51%), grade 4–26 (34%)) and 60 oligodendrogliomas (WHO CNS grade 2–21 (35%), grade 3–39 (65%)) (Table 1). Age at diagnosis was significantly higher in oligodendroglioma compared to astrocytoma patients (median: 35 vs. 43 years, p 0.0006, Wilcoxon). Calibrated scores from the Heidelberg brain tumor classifier for the methylation subclass of IDH mutant glioma reached 0.99 in median for both glioma subtypes [6]. 22% of astrocytomas and 7% of oligodendrogliomas, respectively, had an unmethylated MGMT promoter (*p* 0.0156, Fisher’s exact test). Furthermore, recurrent tumors were more frequent among oligodendrogliomas (*p* 0.0430, Fisher’s exact test). For 3% of oligodendrogliomas, information about brain tumor classifier results was inconclusive, but tumors showed a clear 1p/19q-codeletion in methylome-based copy number profiling and were therefore classified as oligodendrogliomas.

**Table 1:** Description of the main study cohort

	Astrocytoma (*n* = 77)	Oligodendroglioma (*n* = 60)	*P* value
Epidemiological and clinical parameters			
Gender n (%)	Male 48 (62), Female 29 (38)	Male 36 (60), Female 24 (40)	0.8601^a^
Age at diagnosis median (range) in years	35 (18–62)	43 (16–70)	0.0006^b^
Primary tumor n (%)	63 (83)	40 (67)	0.0430^a^
KPS prior to surgery median (range)	100 (70–100)	100 (20–100)	0.2650^b^
Extent of resection n (%)			0.6901^c^
Total	16 (27)	11 (25)	
> 90%	12 (20)	6 (14)	
< 90%	9 (15)	10 (23)	
Biopsy only	22 (37)	17 (39)	
Treatment n (%)			0.3617^c^
Radiation + Chemotherapy	53 (69)	36 (62)	
Resection only	13 (17)	7 (12)	
Radiation only	4 (5)	7 (12)	
Chemotherapy only	2 (3)	4 (7)	
Missing data or no treatment	5 (6)	4 (7)	
Observation prior to resection n (%)	na/0 days 59 (77), 18 (23) median 2257 days (2–8115 days)	na/0 days 41 (68), 19 (32) median 1787 days (21–5619 days)	
Molecular parameters			
V11b4 HD brain tumor classifier methylation class n (%)	Glioma, IDH mutant 77 (100)	Glioma, IDH mutant 58 (97), missing 2 (3)	
V11b4 HD brain tumor classifier calibrated score median (range)	0.99 (0.38–0.99)	0.99 (0.42–0.99)	0.6788^b^
V11b4 HD brain tumor classifier methylation subclass n (%)	Astrocytoma 62 (81), high-grade astrocytoma 15 (19)	Oligodendroglioma 1p/19q-codeleted 56 (93), astrocytoma 2 (3), missing 2 (3)	
MGMT promoter methylation status n (%)	Unmethylated 17 (22), Methylated 57 (74), missing 3 (4)	Unmethylated 4 (7), Methylated 54 (90), missing 2 (3)	0.0156^a^
Histological parameters			
WHO CNS grade 2	12 (16)	21 (35)	
WHO CNS grade 3	39 (51)	39 (65)	
WHO CNS grade 4	26 (34)	na	

Epidemiological, clinical and molecular characteristics

*P* values with numbers in superscript indicating statistical test: ^a^Fisher’s exact test, ^b^Wilcoxon, ^c^Pearson; within “Extent of resection” and “Treatment”, respectively, all categories were considered together for *p* value computation

*P* values highlighted in italic indicate significance

DNA methylation profiling and reference-free deconvolution of 137 gliomas were performed following the protocols described in [25, 34]. Briefly, MeDeCom extracts Latent Methylation Components (LMCs) and LMC proportions across the samples from the input bulk DNA methylation data using non-negative matrix factorization. We identified six LMCs representing major patterns of data variation based on the 5,000 most variably methylated CpGs (lambda 0.001; Supplementary Fig. 1a; Material and Methods). Patients with astrocytoma, CNS WHO grade 4, showed the worst overall survival within the study cohort (Supplementary Fig. 1b).

### LMC-based classification partially overlaps with epigenetically classified glioma subtypes and CNS malignancy grades

**Fig. 1:**
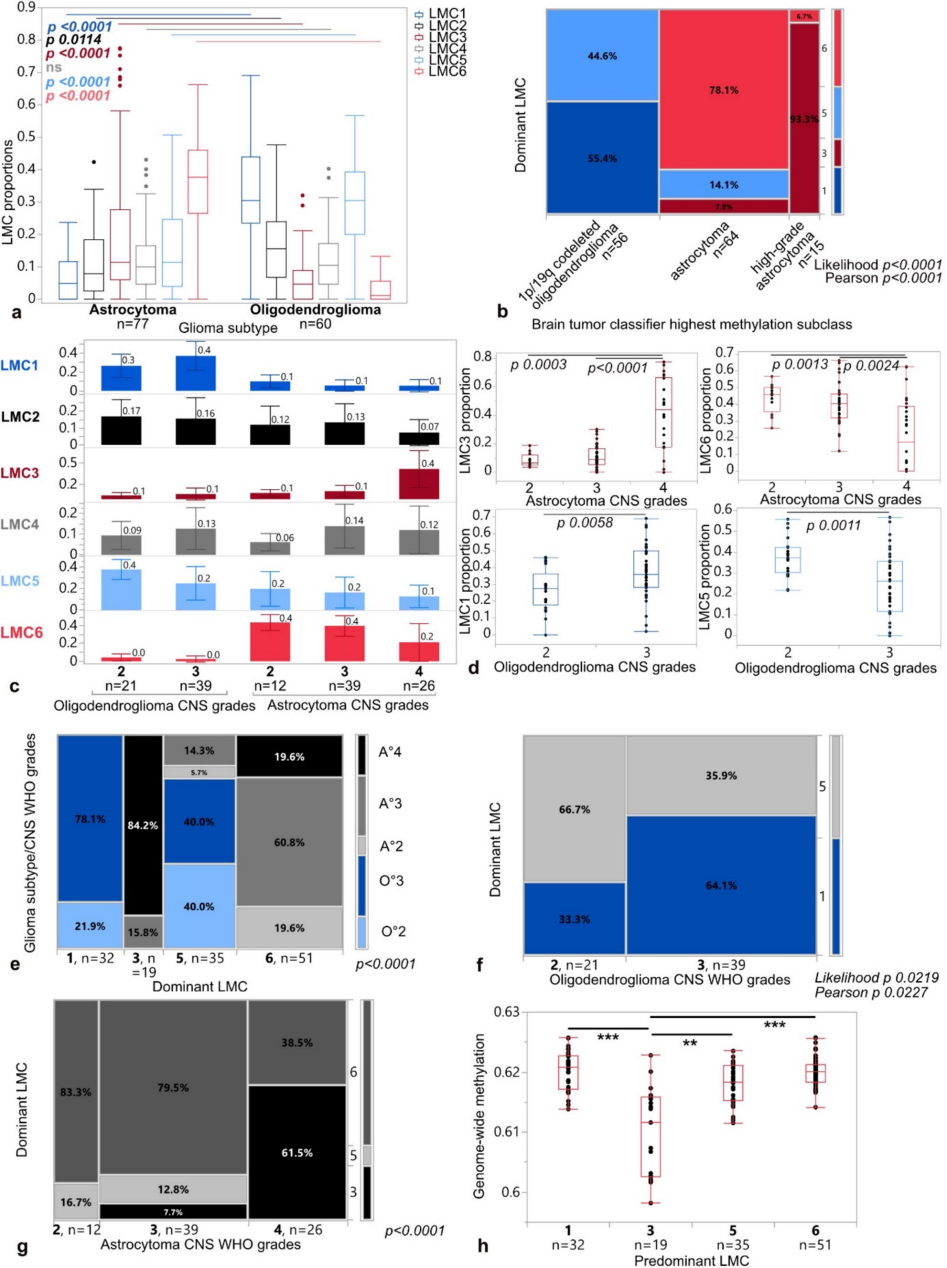
Associations of LMC proportions with glioma subtypes and grades of the training cohort. **a** LMC 1–6 proportions in IDH mutant astrocytomas vs. oligodendrogliomas, box plots. **b** Distribution of frequency of LMC predominancy 1/3/5/6 within tumors assigned to methylation classes “1p/19q-codeleted oligodendroglioma”, “astrocytoma” or “high-grade astrocytoma”, contingency table. **c** Distribution of LMC proportions in gliomas according to subtype “oligodendroglioma” or “astrocytoma” with respect to CNS WHO grades. Box plots with indicated median LMC values. **d** Distribution of LMC proportions 3 and 6 (upper panel) for astrocytoma CNS WHO grades and 1 and 5 (lower panel) for oligodendroglioma CNS WHO grades, box plots. **e** Distribution of frequencies of LMC 1/3/5/6 predominancy within tumors, stratified according to subtypes and CNS WHO grades. **f** Distribution of frequencies of LMC 1/5 predominancy within oligodendrogliomas of all CNS WHO grades. **g** Distribution of frequencies of LMC 3/5/6 predominancy within astrocytomas of all CNS WHO grades. **h** Genome-wide methylation in tumors stratified according to LMC predominancy. *P* values * < 0.05, ** < 0.01, *** < 0.001

LMC proportions within the study cohort showed glioma subtype-specific patterns. While LMCs 3 and 6 were associated with astrocytomas, oligodendrogliomas showed higher proportions of LMC1, 2, and 5 (Fig. 1a). Among epigenetic subclasses of astrocytoma defined by the Heidelberg classifier, high-grade astrocytomas showed significantly higher scores for LMC3 and lower scores for LMC6 compared to the methylation subclass “astrocytoma” (*p* < 0.0001, Likelihood and Pearson; Fig. 1b). Proportions of oligodendroglioma-specific LMC1 were significantly higher in CNS WHO grade 3 than 2 (*p* 0.0058; Fig. 1c, d). Conversely, CNS grade 2 oligodendrogliomas showed higher scores for LMC5 (*p* 0.0011). For astrocytomas of CNS WHO grades 2 and 3, we observed highest LMC6 scores (2 vs. 4 *p* 0.0013; 3 vs. 4 *p* 0.0024), whereas CNS WHO grade 4 astrocytomas were characterized by highest LMC3 scores (4 vs. 2 *p* 0.0003; 4 vs. 3 *p* < 0.0001).

Subsequently, we attributed each glioma to its dominant LMC by assigning it to the LMC with the highest proportion. LMC1-predominant tumors were exclusively oligodendrogliomas, with 78.1% corresponding to CNS WHO grade 3 and 21.9% to grade 2 (Fig. 1e). LMC3-predominancy was exclusive to higher-grade astrocytoma (84.2% CNS WHO grade 4 and 15.8% of grade 3). LMC6-predominant tumors were astrocytomas, with 60.8% of CNS WHO grade 3, and 19.6% of grade 2 and 4, respectively. The LMC5 predominant group was mixed: 40% of CNS WHO grade 2 oligodendrogliomas, 40% of grade 3 oligodendrogliomas, as well as 14.3% of grade 3 and 5.7% of grade 2 astrocytoma. For oligodendrogliomas, dominant LMC patterns were limited to LMC1 and LMC5; higher CNS WHO grade was significantly associated with LMC1-predominancy (*p* 0.0219 Likelihood, *p* 0.0227 Pearson; Fig. 1f). For astrocytomas, we observed that LMC-predominancy depended on CNS WHO grade: The higher the CNS WHO grade in astrocytoma, the more likely tumors clustered within the LMC3-predominant group with lower proportions of LMC5 and 6 (*p* < 0.0001; Fig. 1g). LMC3-dominant tumors furthermore showed a significant reduction in genome-wide methylation levels as compared to all other LMC-dominant tumor groups (Fig. 1h).

### LMC-based grading of glioma is associated with overall survival in a TCGA subgroup of oligodendroglioma patients

We determined a cutoff of 0.39 for the LMC1 proportion predicting the assignment to oligodendrogliomas with copy number alterations in addition to 1p/19q-codeletion in our discovery cohort using recursive partitioning (Supplementary Fig. 1c).

**Fig. 2:**
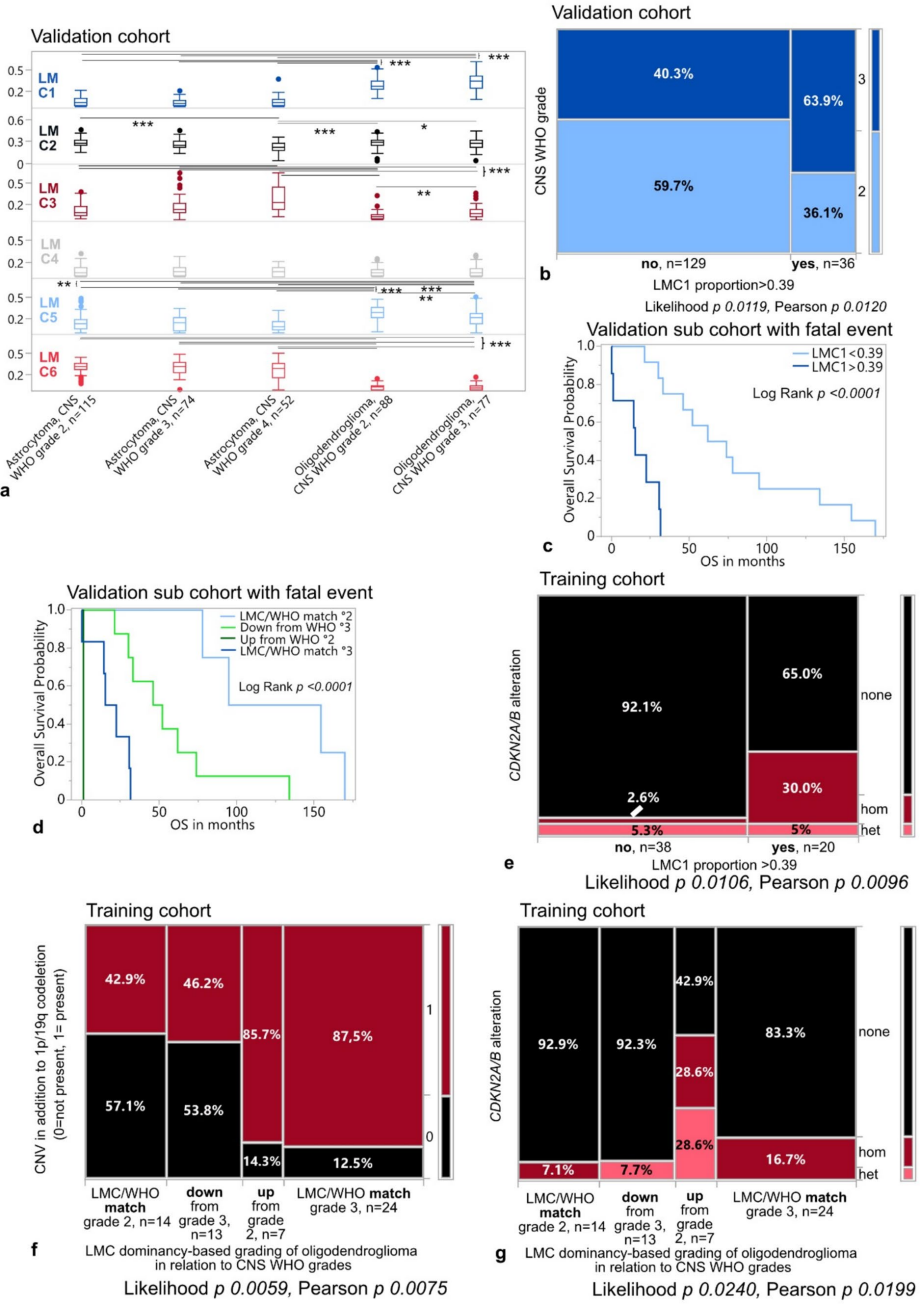
Validation of LMCs and LMC-based grading. **a** Distribution of LMC proportions in TCGA LGGs stratified according to glioma subtype and CNS WHO grade. Box plots with indicated median LMC values. *P* values * < 0.05, ** < 0.01, *** < 0.001. **b** Distribution of frequencies of oligodendrogliomas from the TCGA cohort within categories LMC < and > 0.39, respectively. **c** Kaplan–Meier survival curve for OS in TCGA oligodendrogliomas with event of death (n = 19) dichotomized according to < / > LMC1 = 0.39 (LMC1 < 0.39, *n* = 12; LMC1 > 0.39, *n* = 7). **d** Kaplan–Meier survival curve for OS in TCGA oligodendrogliomas with fatal disease course dichotomized according to LMC1 cutoff-based grading in relation to CNS WHO grades (LMC/WHO match grade 2, *n* = 4; Down from grade 3, *n* = 8; Up from grade 2, *n* = 1; LMC/WHO match grade 3, *n* = 6). **e** Contingency table for frequencies of *CDKN2A/B* copy number alterations “none”, “hom” (homozygous deletion) and “het” (heterozygous deletion) in oligodendrogliomas of the TCGA training cohort stratified according to LMC1 proportion < / > 0.39. **f** Contingency table for frequencies of copy number variations (CNV) in addition to 1p/19q-codeletion in oligodendrogliomas graded by LMC proportions into matching, down- or upgraded samples in comparison to CNS WHO grades. **g** Contingency table for frequencies of *CDKN2A/B* copy number alterations “none”, “hom” (homozygous deletion) and “het” (heterozygous deletion) in oligodendrogliomas graded by LMC proportions into matching, down- or upgraded samples in comparison to CNS WHO grades

To validate the computed patterns of high- vs. low-grade glioma associated LMCs, we transferred them into the TCGA LGG validation cohort comprising 406 tumors with 165 oligodendrogliomas (CNS WHO grade 2 *n* = 88, grade 3 *n* = 77) and 241 astrocytomas (CNS WHO grade 2 *n* = 115, grade 3 *n* = 74, grade 4 *n* = 52; Material and Methods). The relationship between LMCs and glioma subtypes and grades was largely preserved (Fig. 2a). High proportions of LMC1 were associated with oligodendroglioma, CNS WHO grade 3, of LMC5 with oligodendroglioma, CNS WHO grade 2, of LMC3 with astrocytoma, CNS WHO grade 4, and of LMC6 with astrocytoma, CNS WHO grade 2/3 (Supplementary Fig. 1d).

Samples with proportions of LMC1 larger than 0.39 in the TCGA validation cohort were more likely higher- than lower-grade oligodendrogliomas (Fig. 2b). Among patients of the TCGA cohort with a fatal disease course (*n* = 19 of 165) oligodendrogliomas with LMC1 proportions bigger than 0.39 were associated with shorter overall survival (Fig. 2c). Along that line, using LMC-based grading, patients whose tumors were downgraded from a WHO grade 3 showed a more beneficial overall survival than those with a match for grade 3 between WHO- and LMC-based grading as well as those upgraded from a WHO grade 2 (Fig. 2d). While the LMC-based grading helped to identify patients with the worst overall survival among those with least beneficial disease courses, it did not capture differences in overall survival for the whole TCGA cohort oligodendrogliomas after stratification according to LMC1 proportions or LMC-based match/up- or downgrade in relation to WHO grades as compared to WHO-based grading (Supplementary Fig. 1e). Oligodendrogliomas with homozygous deletion of *CDK2NA/B* were significantly enriched within tumors harboring LMC1 proportions higher than 0.39 (Fig. 2e). Samples displaying higher-grade traits based on WHO- or LMC-grading (“LMC/WHO match grade 3” and “up from grade 2”) were more likely to present with additional copy number alterations beyond 1p/19q-codeletion. Oligodendrogliomas with LMC-based grading indicative of a lower-grade glioma (“down from grade 3”) on the contrary, were less likely to show additional copy number aberrations, like “LMC/WHO match grade 2” (Fig. 2f). Along that line, samples displaying homozygous *CDKN2A/B* deletion majorly enriched within the groups “LMC/WHO match grade 3” and LMC-based “up from grade 2” (Fig. 2g).

### Distinct LMCs correlate with general H&E histopathological, aging and contrast agent- enrichment traits of tumors

**Fig. 3:**
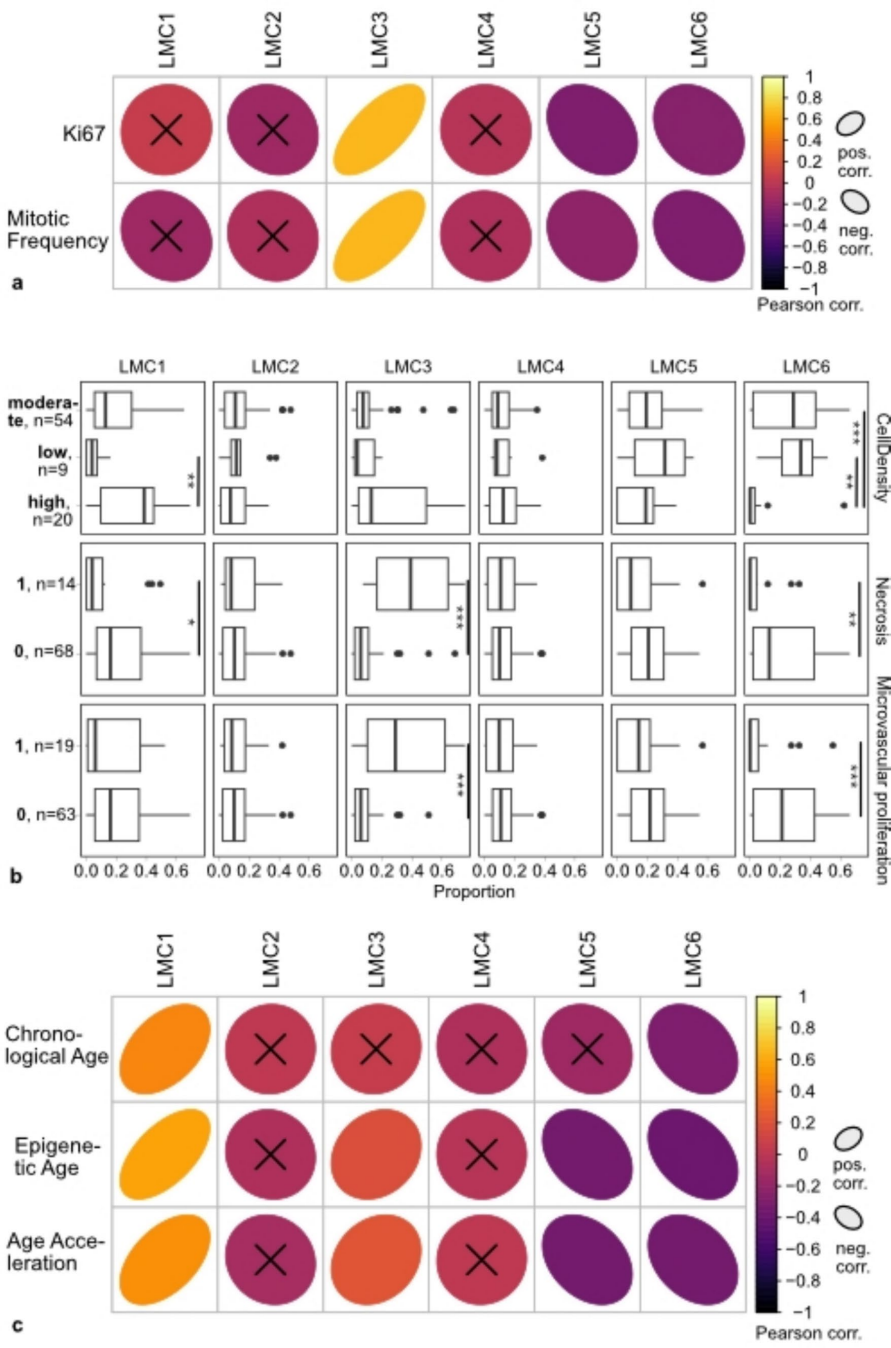
Association of LMCs with markers of tumor proliferation, histological signs of malignancy and age metrics. **a** Correlation of LMC 1–6 proportions with proliferative activity, measured by estimation of Ki67 immunoreactive nuclei and mitotic frequency/mm^2^. Pearson correlation (Pearson corr.) coefficients are color-coded from 1 (yellow, strong positive correlation; ascending ellipse) to −1 (purple, strong negative correlation; descending ellipse). “X” indicates non-significant correlations. **b** Comparison of LMC 1–6 proportions with cellular density (“intermediate”, “low”, “high”), necrosis (present “1”, absent “0”) and microvascular proliferation (present “1”, absent “0”). *P* values * < 0.05, ** < 0.01, *** < 0.001. **c** Correlation of LMC1-6 proportions with patients’ chronological ages, epigenetic ages and DNA methylation age acceleration. Pearson correlation (Pearson corr.) coefficients are color-coded from 1 (yellow, strong positive correlation; ascending ellipse) to −1 (purple, strong negative correlation; descending ellipse) and elliptical shape indicate stronger correlation. “X” indicates non-significant correlations

LMC3 correlated significantly with increased mitotic and proliferative activity, the latter measured by Ki67 immunoreactive nuclei, whereas LMC5 and 6 showed a significant negative correlation with mitotic frequency and proliferation (Fig. 3a). LMC1, 2, and 4 were not significantly associated with mitotic frequency or proliferative activity.

LMC3 was significantly associated with the presence of necrosis and microvascular proliferation as histopathological signs of malignancy in tumors (*p* < 0.0001; Fig. 3b). High proportions of LMC1 were associated with elevated tumor cell density and absence of necrosis (*p* 0.0038 and 0.0167, respectively). Tumors with high LMC6 proportions were less likely to present with high cellularity, microvascular proliferation, or necrosis (*p* 0.0022, 0.0002, 0.0006 and 0.0011, respectively). LMC2, LMC4, and LMC5 were not significantly associated with any histological markers of malignancy. LMC3-predominant tumors showed enrichment of contrast agent in MRI scans as a marker of higher-grade glioma in up to 80% of cases. In contrast, LMC5-predominant tumors presented without contrast agent enrichment in 76.7% (Supplementary Fig. 2a). The assignment of predominant LMCs to gliomas was independent of patient sex (Supplementary Fig. 2b).

Next, we checked for association of LMC proportions with aging parameters as age is an important risk factor for higher grade gliomas and the subtypes of IDH mutant glioma were shown to differ in terms of DNA methylation age acceleration [9, 22, 42]. DNA methylation age acceleration is computed by subtraction of the patients’ chronological ages from epigenetic ages derived from the tumor methylomes using a methylclock [28]. While LMC1 proportions in tumors correlated positively with chronological patient ages, there was a negative correlation between LMC5 and 6 and age (LMC1 R^2^ corr. 0.2, *p* < 0.0001; LMC5 R^2^ corr. 0.017, *p* 0.071; LMC6 R^2^ corr. 0.084, *p* 0.0003; Fig. 3c). Similarly, we observed an association between LMC1 and epigenetic aging (R^2^ corr. 0.39, *p* < 0.0001). Higher LMC6 scores on the contrary correlated negatively with epigenetic age (R^2^ corr. 0.23, *p* < 0.0001). Along that line, tumors with high LMC1 proportions were characterized by increased DNA methylation age acceleration while high LMC6 proportions in glioma correlated with DNA methylation age deceleration (LMC1 R^2^ corr. 0.18, *p* < 0.0001; LMC6 R^2^ corr. 0.14, *p* < 0.0001). The other LMCs were not significantly associated with chronologic or epigenetic age metrics. In general, oligodendrogliomas of higher malignancy grade showed higher epigenetic age compared to astrocytomas, whilst chronological patient age exclusively differed between oligodendroglioma and astrocytoma of grade 3 (Supplementary Fig. 2c). DNA methylation age acceleration however was significantly increased in oligodendroglioma grade 3 in comparison to astrocytomas of all WHO CNS grades. LMC1-dominant tumors showed highest chronological and epigenetic ages. Chronological and epigenetic age were significantly associated with LMCs 5 and 6, and epigenetic age was significantly higher in LMC1- than LMC3-dominant tumors (Supplementary Fig. 2d). LMCs 1 and 5 showed significantly higher DNA methylation age acceleration in comparison to LMC3 and 6. Most oligodendrogliomas in our training cohort were initially diagnosed after neurosurgical resection, but for some tumor growth was observed for a longer time period (median 1787 days). Within the latter subgroup of oligodendroglioma, the increase of epigenetic age depended on CNS WHO grade majorly but not on the period of observed tumor growth (Supplementary Fig. 2e).

### LMC1 and LMC3 correlate positively with tumor cell content

**Fig. 4:**
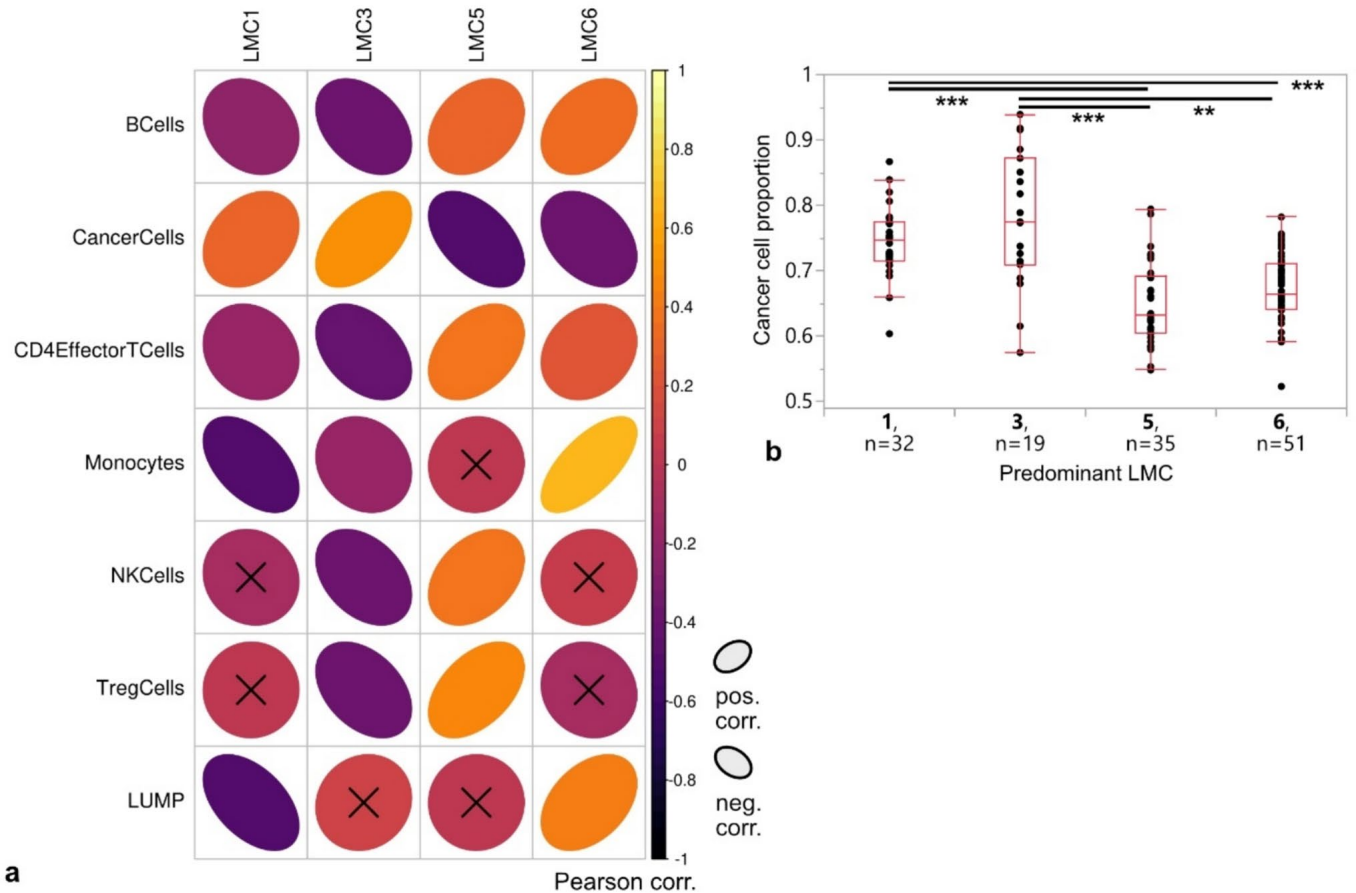
Association of LMC proportions with cellular composition of gliomas. **a** Correlation of LMCs 1, 3, 5, and 6 with estimates of immune cells (B cells, CD4 effector T cells, NK cells and T regulatory cells), monocytes, cancer cells, and LUMP. Pearson correlation (Pearson corr.) coefficients are color-coded from 1 (yellow, strong positive correlation; ascending ellipse) to −1 (purple, strong negative correlation; descending ellipse) and elliptical shape indicates stronger correlation. “X” indicates non-significant correlations. **b** Distribution of cancer cell estimates in tumors stratified according to LMC predominancy, box plots. *P* values * < 0.05, ** < 0.01, *** < 0.001

LMCs were subjected to DNA methylation-based, reference-based tumor deconvolution to infer their cellular composition [8, 17]. LMC1 and LMC3, which were rather associated with higher-grade tumors, showed significant positive correlation with cancer cell proportions (LMC1 R^2^ corr. 0.070, *p* 0.011; LMC3 R^2^corr. 0.26, *p* < 0.0001; Fig. 4a) whereas correlations with immune cell subsets and monocytes were negative. Higher scores for LMC5 and LMC6, on the other hand, correlated negatively with cancer cell proportions (LMC5 R^2^ corr. 0.22, *p* < 0.0001; LMC6 R^2^ corr. 0.13, *p* < 0.0001) but positively with B and T cell subsets. Notably, LMC6 proportions positively correlated with predicted monocyte content (R^2^ corr. 0.45, *p* < 0.0001). Along this line, we employed the LUMP algorithm to infer overall leukocyte proportions in LMCs. LMC1 correlated negatively with LUMP proportions, while higher LMC6 proportions coalesced with higher LUMP scores (LMC1 R^2^ corr. 0.23, *p* < 0.0001; LMC6 R^2^ corr. 0.16, *p* < 0.0001). The estimated cancer cell proportions for LMC1- and LMC3-dominant tumors did not differ between each other but were significantly higher than those computed for the lower-grade glioma-associated LMCs 5 and 6 (Fig. 4b).

### High-grade LMCs 1 and 3 show G-CIMP characteristics

**Fig. 5:**
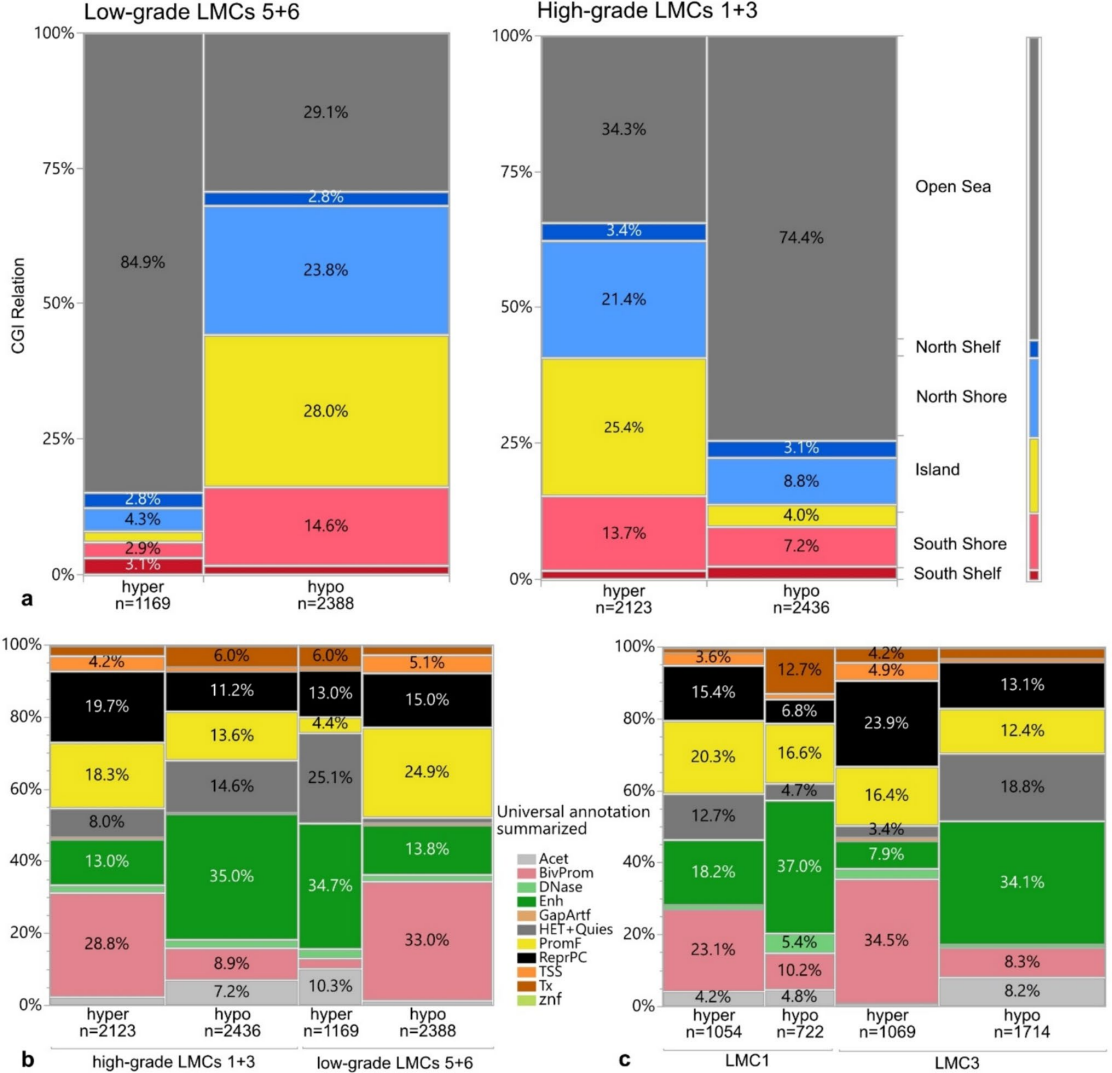
Epigenetic backbone of LMCs. **a** Distribution of frequencies of hyper- or hypomethylation with respect to CGI relation in low-grade vs. high-grade LMCs. **b** Distribution of hyper- and hypomethylation frequencies with respect to summarized chromatin landscape universal annotation in high-grade vs. low-grade LMCs. **c** Location of hyper- and hypomethylated CpGs with respect to different chromatin states comparing high-grade LMCs associated with oligodendroglioma (LMC1) vs. astrocytoma (LMC3). Values are displayed in % of hyper- or hypomethylation separately within each LMC category. Human genome regions associated with the following states/traits according to Universal Annotation [38]: *Acet* Acetylated chromatin, *BivProm* Bivalent Promoters, *DNase* DNase I hypersensitivity, *Enh* Enhancers, *GapArtf* Assembly gaps and alignment artifacts, *HET* Heterochromatin, *Quies* Quiescent chromatin, *PromF* Promoter flanking, *ReprPC* Polycomb repressed chromatin, *TSS* Transcriptional start sites, *Tx* Exons and transcription, *znf* Zinc finger

We next set out to analyze the differential epigenetic backbone of LMC composition in high- vs. low-grade associated LMCs. To that end, we performed differential analysis between each LMC compared to all other LMCs and defined LMC-specific hypo- and hypermethylated CpGs (Material and Methods). Sites hypermethylated in LMCs 5 and 6 were mainly located outside of CpG islands (termed ‘open sea’; Fig. 5a). Conversely, CpGs hypomethylated in LMC5 and 6 were preferentially located within and in proximity to CpG islands. This pattern was reversed in high-grade glioma-associated LMCs 1 and 3 as they harbored hypomethylation mainly in open sea regions and hypermethylation in chromatin regions mapping within the north shore, CpG island, and south shore.

In higher-grade LMCs 1 and 3, 28.8%, 19.7%, and 18.3% of hypermethylated CpGs were located in bivalent promoters (BivProm), sites repressed by polycomb (ReprPC) and promoter-flanking chromatin regions (PromF), whereas hypermethylated CpGs in lower-grade LMCs 5 and 6 mapped with enhancers (Enh; 34.7%), hetero- and quiescent chromatin (HET + Quies; 25.1%), polycomb-repressed sites (ReprPC; 3%) and acetylated chromatin regions (Acet; 10.3%; Fig. 5b).

Hypomethylation at enhancers and hypermethylation at bivalent promoters was one of the major patterns found in both high-grade LMCs (Supplementary Fig. 3a, b). When compared to LMC1, LMC5 composition was characterized by more hypermethylated CpGs mapping with hetero- and quiescent chromatin and enhancers as well as hypomethylated sites located within bivalent promoters and promoter flanking CpGs (Supplementary Fig. 3c). These results were similar for the comparison between the composition of astrocytoma-associated LMC 6 vs. 3 (Supplementary Fig. 3d). Overall, LMC1 was defined by significantly more hypermethylated sites than LMC5 (Supplementary Fig. 3e). The number of hypo- and hypermethylated sites forming LMC3 and 6 was balanced (Supplementary Fig. 3f). Between the glioma subtype-specific LMCs, there was majorly no overlap of CpGs (Supplementary Fig. 3g, h).

## Discussion

Epigenetic profiling became an indispensable component for a comprehensive evaluation of brain tumors in neuropathological diagnostics. Classification tools, like the Heidelberg brain tumor classifier, are based on the robustness of tumor-type-specific DNA methylation patterns. Deconvolution analysis, on the other hand, allows for an unsupervised analysis of DNA methylation data, facilitating the discovery of novel tumor-associated methylation patterns irrespective of additional confounding factors [14, 34].

In 137 IDH-mutant gliomas, we showed differences between IDH mutant glioma subtypes on methylation level similar to LGm1-3 groups defined by Cecarrelli et al. [7]. LMCs were exclusively associated with either astrocytoma or oligodendroglioma, 1p/19q-codeleted. LMC analysis, however, reflected histological parameters of high- vs. low-grade gliomas, which were not apparent upon separation of LGm1/LGm2 associated with 1p/19q-intact and LGm3 1p/19q-codeletion-enriched methylome-based clusters [7]. LMC3 defined a high-grade astrocytoma LMC, with features such as increased mitotic frequencies, while LMC6 defined a low-grade astrocytoma DNA methylation subtype. Similarly, CNS WHO grade 2 oligodendrogliomas presented with higher proportions of LMC5, whereas CNS WHO grade 3 tumors harbored higher ratios for the oligodendroglioma-specific LMC1. Changes of LMC proportions in glioma might thus reflect their malignant transition from lower- to higher-grade astrocytoma and oligodendroglioma, respectively.

We observed hypermethylation in high-grade LMCs compared to low-grade LMCs at and around CpG islands, at the expense of hypermethylation of sites located in open sea regions. Previous work has established a clear link between IDH mutant gliomas and the glioma CpG island hypermethylator (G-CIMP) phenotype [31]. DNA hypermethylation of CpG islands was characterized as a stable phenomenon as it persists in recurrent G-CIMP gliomas independent of the CNS grade [36]. Nevertheless, a prognostically unfavorable genome-wide hypomethylation was shown in recurrent IDH mutant, non-codeleted gliomas [4, 36]. G-CIMP high to low progression overall was found to be a process independent from initial glioma grade leading to a phenotype resembling mesenchymal-like IDH wildtype glioblastoma [36]. Analysis conducted by Mur et al. argues for differences in G-CIMP-related methylation distribution across glioma subtypes because codeleted glioma-CIMP cases showed slightly increased proportions of hypermethylated CpG sites at islands at the expense of open sea regions when compared to non-codeleted glioma-CIMP samples [29]. LMC1- vs. -5- and -6-dominant tumors show comparable overall-genome-wide methylation levels. Nevertheless, the high- and low-grade LMCs differ with regard to cancer cell proportions, which might be an indication that the lower-grade LMC signature is driven by non-neoplastic cellular components of the tumor microenvironment. The fact that the higher-grade LMCs are composed of either oligodendroglioma or astrocytoma but the lower-grade LMC5 is mixed would corroborate this hypothesis. In future experiments, single-cell methylome analysis will be needed to answer how much each single neoplastic and non-neoplastic cell type contributes to each of the LMCs. It is, however, up for discussion whether malignant transition emphasizes a distinct methylation phenotype which is pre-arranged but not yet dominant in lower-grade glioma tumor cells. Since glioma methylomes from bulk tumors are routinely acquired and subjected to classifier tools for diagnostic purposes, there is a clinical need for approaches for refined glioma grading which reflect the common tissue and data type; the inclusion of tumor microenvironmental traits for tumor classification thereby is mandatory.

We observed an enrichment of hypermethylation at bivalent promoters in high-grade LMCs, which are associated with genes involved in cell differentiation and development in embryonic stem cells. Bivalent genes are often targeted by aberrant DNA methylation in human cancers while being hypomethylated in non-neoplastic cells, like embryonic stem cells [21, 24, 39, 41]. DNA methylation-induced irreversible gene silencing of bivalent genes is shown to be leveraged by a resolution of their histone modification hallmark pattern, the H3K4me3/H3K27me3-double marking, which might give rise to cellular adaptation and epigenetic plasticity thereby fueling malignant behavior in gliomas [21, 26]. Tissue-specific bivalent gene expression in development is furthermore shown to be orchestrated via a synergistic effect between bivalent promoters and active enhancers marked by H3K27ac and therefore exhibiting an accessible chromatin conformation [10, 41]. Potentially, hypomethylation of enhancers as observed in higher-grade LMCs might contribute to a chromatin configuration which facilitates gene expression. Since in higher-grade astrocytoma it is shown that although developmental genes gained DNA hypermethylation, their expression was increased instead of silenced, bivalent genes might potentially benefit from aberrant enhancer activation thereby bringing higher order chromatin structure into play as a mechanism facilitating malignant glioma transition [15].

Oligodendrogliomas stood out by showing the highest epigenetic ages. Epigenetic aging and DNA methylation age acceleration recently emerged as potential biomarkers in diffuse glioma; however, underlying mechanisms and consequences are far from being well understood. Increased epigenetic aging has already been described for oligodendroglioma, IDH mutant and 1p/19q-codeleted, in comparison to IDH wildtype gliomas and IDH mutants without codeletion, especially when the Horvath pan-age and pan-tissue methylclocks were used for epigenetic age computation [22, 42]. By use of a mitosis-related methylclock for epigenetic age estimation, epiTOC, the authors found the classic subtype of glioblastoma to also be associated with increased aging on an epigenetic level. Along that line, and in IDH wildtype glioblastoma, we and others found positive effects of tumors with increased epigenetic aging on patient survival [3, 5]. This study, however, argues for a more careful interpretation of the biomarker epigenetic aging in IDH mutant glioma because WHO CNS grade 3 oligodendrogliomas, as well as LMC1-dominant tumors, showed higher age metrics than their WHO CNS grade 2 counterparts and LMC5-dominant tumors. Since higher chronological age is associated with higher CNS WHO grade in oligodendroglioma, it is challenging to discriminate between the effects imposed on epigenetic aging by higher-grade tumor biological processes vs. duration of in vivo tumor growth [9]. Arguing for methylation changes due to higher-grade tumor-specific intrinsic processes, however, is the fact that in our cohort, epigenetic ages did not differ with regard to in vivo growth duration (longer or shorter than the cohort median of 1787 days for oligodendrogliomas) but majorly to CNS WHO grades. This suggests that an increase in epigenetic ages is accompanied by a higher-grade phenotype in oligodendroglioma.

We identified LMC1 as the methylome-based equivalent of high-grade oligodendroglioma. Unlike LMC3-dominant tumors, tumors scoring high for LMC1 did not show apparent histological signs of malignancy in that there was no association with microvascular proliferation or necrosis. Figarella-Branger et al., who studied roughly 500 patients suffering from oligodendroglioma, stratified the tumors according to brisk mitotic activity of > / = 2.5 mitotic figures/mm^2^ and presence of necrosis and/or microvascular proliferation. They observed a 91% 5-year OS in group 1 harboring> / = 2.5 mitoses/mm², vs 85% in group 2 harboring microvascular proliferation, and 77% in group 3 harboring microvascular proliferation and necrosis [11]. An advantage of our LMC-based grading approach is the independence from sampling of the most malignant tumor hotspot. Furthermore, LMC-based grading reflected changes of copy number, in that it stratified oligodendrogliomas according to present copy number variations in addition to 1p/19q-codeletion as well as homozygous deletion of *CDKN2A/B*. While current data support the understanding of a negative impact on overall survival when *CDKN2A/B* is homozygously deleted, this was not shown for heterozygous *CDKN2A/B* deletions [1, 19]. It is intriguing that LMC-based grading separated the oligodendrogliomas according to homo- but not heterozygous *CDKN2A/B* deletion status, except for a single grade 2 tumor not meeting any malignancy criterium besides the homozygous *CDKN2A/B* deletion. Future research is needed to explore the prognostic value and biomarker capacity of LMCs in oligodendroglioma in view of distinct tumor cell-specific epigenetic alterations promoting malignant behavior prior to apparent histological or radiological signs of higher-grade glioma.

Also prone to sampling bias is mitotic frequency, which can furthermore be observer dependent. Giannini et al. tested the prevalence, correlation coefficients, and consensus rates of histological malignancy features in 124 lower-grade gliomas. They showed that even though mitotic activity belonged to traits with moderate or substantial interobserver reproducibility, it was not associated with cause-specific survival, which might have been caused by missing neuropathological expertise in one of two rater groups [16]. In the hands of a trained neuropathologist, however, the mitotic count was assumed to mirror cause-specific survival differences, and the authors suggested a cutoff of 2.5 mitoses/mm^2^. Most comprehensive subsequent studies focused on the analysis of clinical and histological parameters in grade 3 oligodendroglioma only [11, 12, 23]. The stratification into three groups (> 2.5 mitoses/mm^2^, angiogenesis present, necrosis present with or without microvascular proliferation) by Figarella-Branger was accompanied by an increasing mitotic count but still did not reflect on OS in multivariate analysis [11]. The lack of a firm definition of a mitotic count cutoff associated with malignancy in oligodendroglioma attenuates our finding of a missing correlation between mitotic count and LMC1 proportions, especially as malignancy features might co-occur in oligodendroglioma. Whether this implies a lower reliability of the biomarker mitotic count for grading needs further testing but warrants a more detailed CNS WHO grade discriminative analysis.

In summary, we propose an epigenetically based support of IDH mutant glioma objectifying current grading strategies relying on morphological tumor traits mostly. For oligodendroglioma, LMC1 was associated with higher grade glioma morphology, CNS grade 3, alterations of the *CDKN2A/B* locus, and chromosomal aberrations in addition to 1p/19q-codeletion. Along that line, patients with tumors presenting with a LMC1 cutoff bigger than 0.39 showed the most unfavorable disease courses among those with registered events of death in comparison to those with cutoffs smaller than 0.39. On an epigenetic level, higher LMC1 proportions were related to hypermethylation at bivalent promoters and promoter-flanking CpG sites, as well as to hypomethylation at enhancers when compared to LMCs associated with lower grade gliomas, delineating potential mechanisms of malignant transition in gliomas. Although we were able to prove that LMC signatures are transferable between oligodendroglioma cohorts, further validation is required. This cutoff would have to stand up to tumor heterogeneity and ideally be able to indicate the very beginning or a critical methylation status switch in glioma evolution towards malignancy, thereby questioning especially this cutoff’s minimum. Future investigations of single-cell methylomes, as well as an increase in case numbers from multicenter cohorts, will target these requirements, ultimately leading to an improvement in oligodendroglioma grading. From a patho-mechanistical point of view, targeted and more expansive analyses of promoter-flanking CpGs, enhancers, and bivalent promoters are needed to elucidate their role in the malignant transition of oligodendroglioma. Since, analogous to LMC1, clear cutoffs for LMC3 are still missing, and our study focused on oligodendroglioma, the discrimination between grade 2 and 3 astrocytomas stays challenging as for now and awaits further in-depth investigation. Nevertheless, distinct methylation gains and losses shaping the understanding of epigenetic patterns of higher grade IDH mutant glioma were partially also encountered in lower-grade astrocytoma, potentially defining the initiation of a malignant transition by the acquirement of LMC3 proportions, which otherwise were mostly associated with grade 4 astrocytoma.

## Supplementary Information

Below is the link to the electronic supplementary material.

Supplementary file1 (XLSX 66 KB)

Supplementary file2 (DOCX 1174 KB)

## Data Availability

All data relevant to the study, analyzed and/or generated within this study are included in the article or uploaded as supplementary information. All other data and raw data IDAT files will be made accessible upon reasonable request.
