# Supplementary material for: DNA methylation analysis reveals an epigenetic signature distinctive of high-grade oligodendroglioma
Source: Acta Neuropathol. 2025 Aug 23;150(1):21. doi: 10.1007/s00401-025-02926-y (PMC12374884; doi:10.1007/s00401-025-02926-y)
Supplement: Supplementary file 2 — Supplementary file2 (DOCX 1174 KB) [file 401_2025_2926_MOESM2_ESM.docx]

**Supplementary Material**


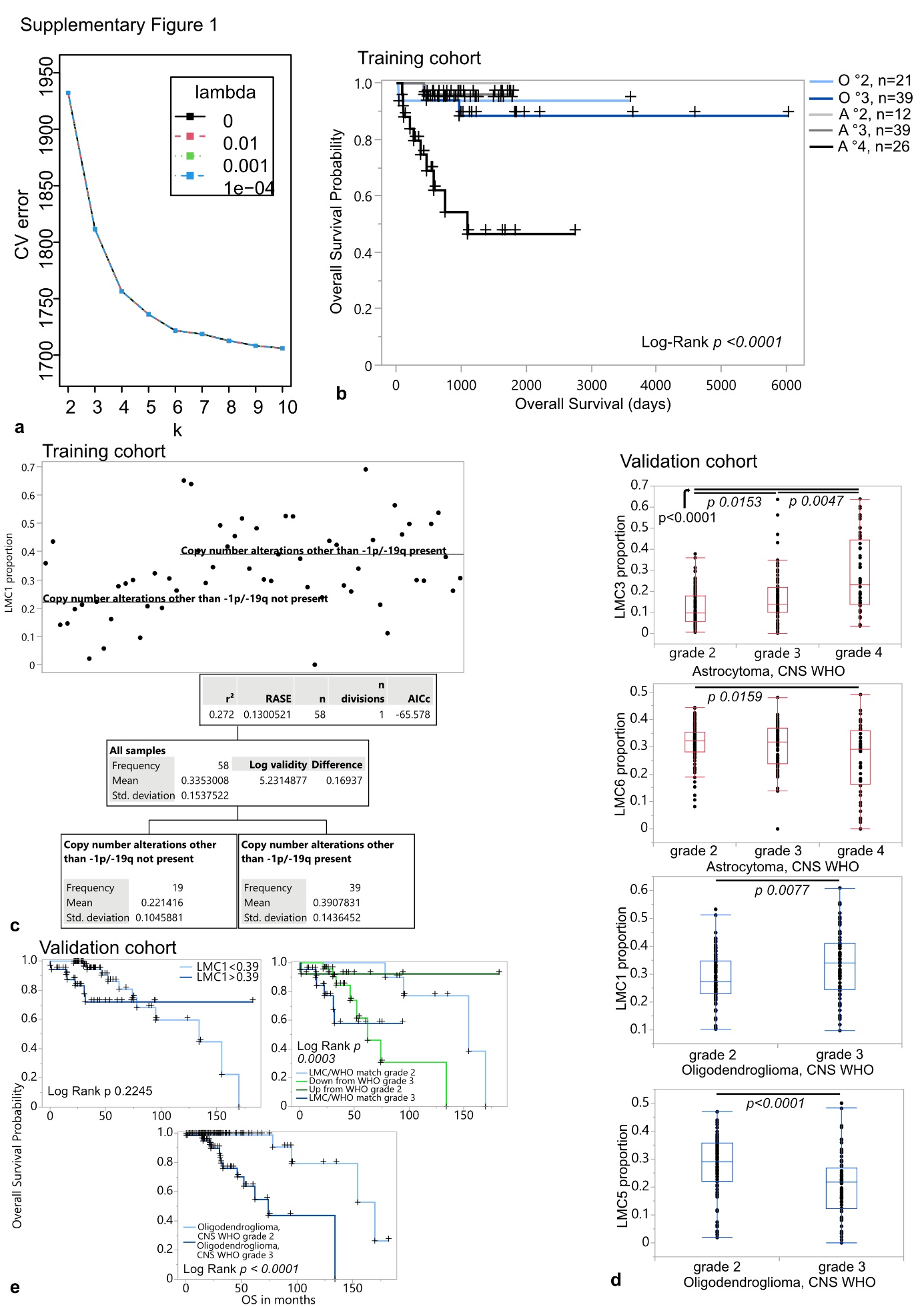


**Supplementary Figure 1:** a) K selection plot. b) Kaplan Meier survival curve for OS in the training cohort dichotomized according to glioma subtype and CNS WHO grade. “+” censored. c) Recursive partitioning model for LMC1 proportions as predictor of the dependent variable “no copy number variation (CNV) in addition to 1p/19q” vs. “present CNV in addition to 1p/19q or unknown” in the training cohort. d) Distribution of LMC proportions 3 (first panel) and 3 (second panel) for astrocytoma CNS WHO grades as well as LMC 1 (third panel) and 5 (fourth panel) for oligodendroglioma CNS WHO grades in the validation cohort, box plots. e) Kaplan Meier survival curves for OS in oligodendrogliomas from the TCGA cohort, stratified according to LMC1 proportion >/<0.39 (upper left panel), LMC1 cutoff-based grading in relation to CNS WHO grades (upper right panel) and CNS WHO grades (lower panel). “+” censored.


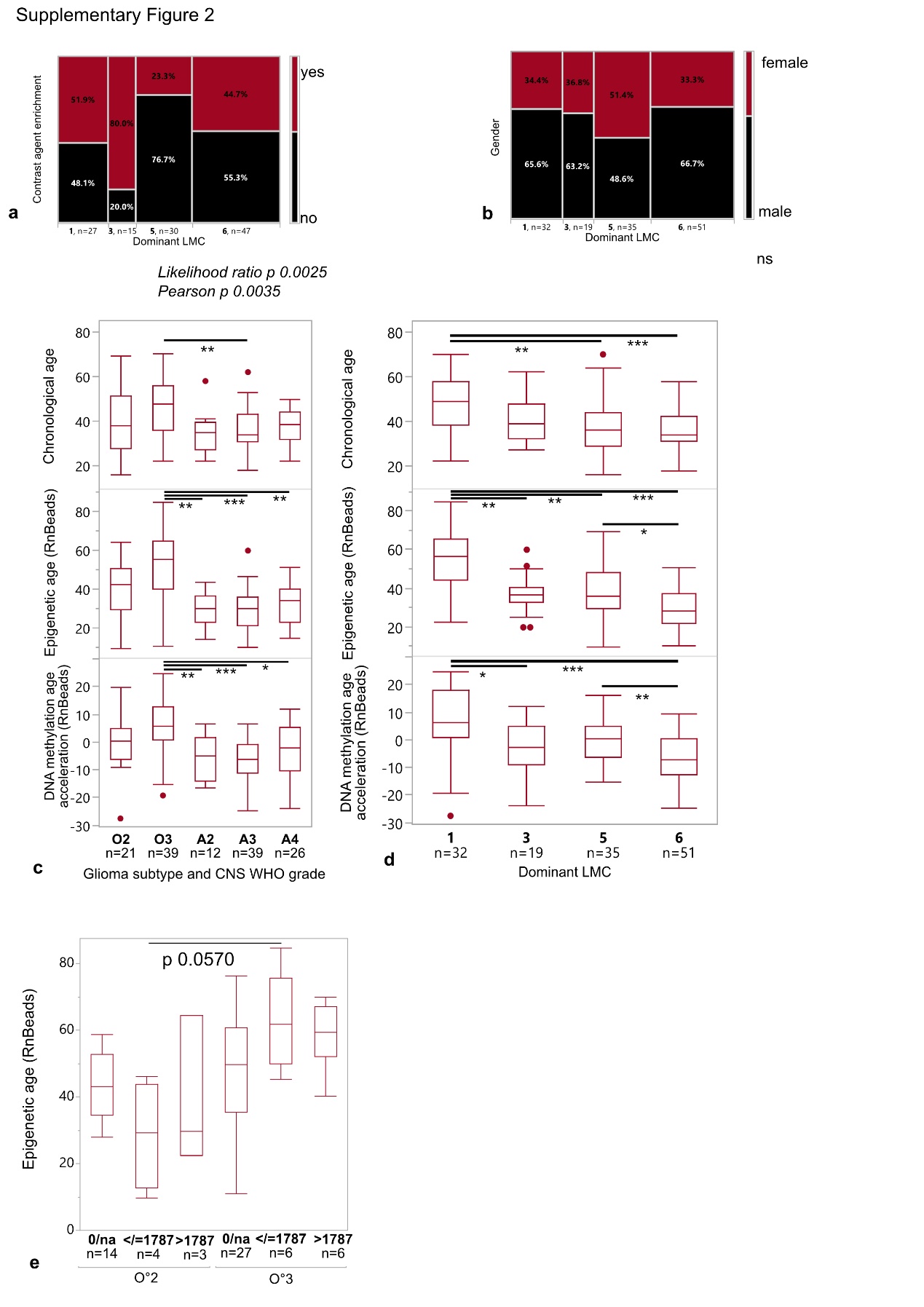


**Supplementary Figure 2:** a) Contingency table of frequency of contrast agent enrichment, categorized in “yes” present vs. “no” absent in tumors assigned to LMC-dominant groups. b) Contingency table of frequency of sex, categorized in “male” and “female” in tumors assigned to LMC-dominant groups. c) Box plots presenting age metrics (chronological age, epigenetic age and DNA methylation age acceleration) in gliomas stratified according to subtypes and CNS WHO grades. d) Box plots presenting age metrics (chronological age, epigenetic age and DNA methylation age acceleration) in gliomas stratified according to predominant LMC. e) Box plots presenting epigenetic ages in oligodendrogliomas, stratified according to CNS WHO grades and period of tumor observation (0/na, </=1787 days, >1787 days). P values * <0.05, **<0.01, ***<0.001.


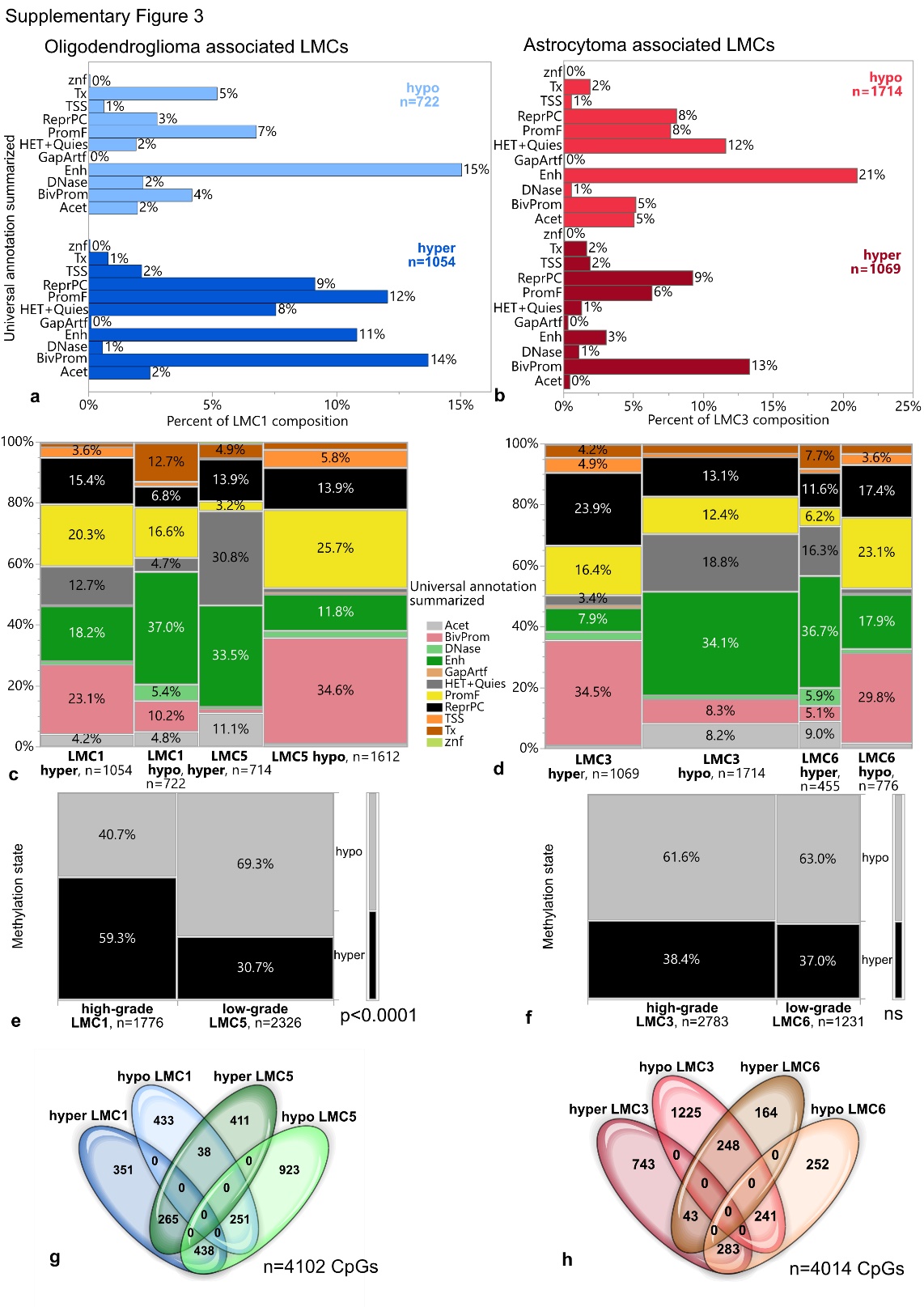


**Supplementary Figure 3:** a) Composition of LMC1 with respect to hyper- and hypomethylation frequencies of CpGs mapped to summarized chromatin landscape universal annotation. b) Composition of LMC3 with respect to hyper- and hypomethylation frequencies of CpGs mapped to summarized chromatin landscape universal annotation. c) Distribution of frequencies of hyper- or hypomethylation with respect to summarized chromatin landscape universal annotation in low-grade vs. high-grade oligodendroglioma-associated LMCs. d) Distribution of frequencies of hyper- or hypomethylation with respect to summarized chromatin landscape universal annotation in low-grade vs. high-grade astrocytoma-associated LMCs. e) Contingency table for distribution of frequencies of hyper- vs. hypomethylated CpG sites in low- vs. high-grade LMCs in oligodendroglioma. f) Contingency table for distribution of frequencies of hyper- vs. hypomethylated CpG sites in low- vs. high-grade LMCs in astrocytoma. g) Venn diagram displaying LMC1- and LMC5-private vs. shared CpG site numbers, respectively, stratified according to methylation status. h) Venn diagram displaying LMC3- and LMC6-private vs. shared CpG site numbers, respectively, stratified according to methylation status. Abbreviations: Human genome regions associated with the following states/traits according to Universal Annotation [38]: *Acet* Acetylated chromatin, *BivProm* Bivalent Promoters, *DNase* DNase I hypersensitivity, *Enh* Enhancers, *GapArtf* Assembly gaps and alignment artifacts, *HET* Heterochromatin, *Quies* Quiescent chromatin, *PromF* Promoter flanking, *ReprPC* Polycomb repressed chromatin, *TSS* Transcriptional start sites, *Tx* Exons and transcription, *znf* Zinc finger.

**Supplementary Data Table (excel)**
